# Growing dominance and soft power enhancement: Exploring Chinese cities’ continual efforts to promote international sports events from a city network perspective

**DOI:** 10.1371/journal.pone.0311171

**Published:** 2024-11-20

**Authors:** Yubin Ou, Gengzhi Huang, Yixiao Xu, Anan Xie, Desheng Xue

**Affiliations:** 1 School of Geography and Planning, Sun Yat-sen University, Guangzhou, Guangdong, P. R. China; 2 Southern Marine Science and Engineering Guangdong Laboratory, Zhuhai, Guangdong, P. R. China; 3 Zhuhai Xingge City Development Co., Ltd., Zhuhai, Guangdong, P. R. China; East China Normal University, CHINA

## Abstract

While studies on World City Networks (WCNs) based on International Sports Events (ISEs) have revealed a nonwesternization evolutionary trend, few studies have focused on high-level ISEs’ long-term spillover effects on promoting nonwestern host countries. This paper aims to fill these gaps by exploring the external connections of Chinese cities in each Olympic Games since 2008 deploying social network analysis and community detection methods. The results show that gateway events, such as the 2008 Beijing Summer Olympic Games and the 2022 Winter Olympic Games, rather than gateway cities, play essential roles in promoting Chinese cities’ status in sports diffusion. Specifically, after the successful hosting of Olympic games, international sports federations (ISFs) witnessed Chinese cities’ ability to organize ISEs; consequently, ISFs would try to select Chinese host cities considering China’s rapid development and the relatively lower popularity of certain sports domestically. Additionally, as ISEs offer significant opportunities for city marketing, many Chinese cities have incorporated hosting ISEs as important urban development strategies. The promotion of the "events globalization" strategy by ISFs and the attraction measures taken by Chinese city governments for ISEs have ultimately led to a continuous increase in the number of Chinese cities and their direct external connections in ISE-based WCNs. This finding fully reveals the long-term spillover effect of high-level ISEs on promoting cities in nonwestern host countries. This further indicates that the nonwesternization trend in ISE-based WCNs may be because an increasing number of high-level ISEs, such as the Olympics and the World Cups, were held in nonwestern countries.

## 1 Introduction

Castells’s theory of the "space of flows" argues that flows of capital, people, and information constantly move between cities, leading to the formation of city networks [1]. Core city nodes in various city networks have the ability to dominate most of the resources in the network [2,3]. Therefore, enhancing the status of cities in world city networks (WCNs) through various strategies has become an important means of gaining leverage for the development of cities (especially those from the Global South) worldwide [4,5].

Among those countries and cities, Chinese cities have continually implemented this strategy and have achieved tremendous success [4,6]. In terms of the economy, in the GaWC ranking based on APS firms, the number of Chinese cities rated B- or above increased from 2 in 2000 to 19 in 2020 [3,7], making China one of the dominant regions in the network. In the political realm, Chinese cities have also been enhancing their political influence through the establishment and integration of various nongovernmental organizations [8,9]. Derudder has also argued that China-led globalization has become one of the three forms of globalization [7]. However, against the backdrop of increasing global cultural dissemination [10], the evolution of the status of Chinese cities in WCNs in the cultural dimension has received little attention from scholars.

In essence, enhancing the influence of cities and countries in the cultural dimension has long been an important national strategy in China [6,11]. Research indicates that Chinese cities generally employ two strategies to enhance their status in cultural dissemination. First, some cities develop well-known cultural production districts or facilities (e.g., Hengdian World Studios, various music districts) [12,13], attracting numerous cultural and creative enterprises and individuals [14]. However, owing to the late development of China’s cultural industry [11], the country’s position in production networks of cultural products such as film, music, and art is far inferior to that of Western countries [13,15,16]. Additionally, the consumption networks of domestic cultural products are mostly confined to China and its neighbouring regions [17,18]. Therefore, this strategy’s impact on enhancing the cultural soft power of cities is mostly limited to the domestic level. Second, some cities may host ’mega events’ or ’global cultural events’ [6,19]. In essence, ’global cultural events’ are landmark events in global cultural dissemination [20]. High-level cultural events not only position cities as important nodes in global cultural dissemination [21] but also provide a tremendous platform for city marketing and image shaping [6,19], thereby enhancing the city’s global recognition and cultural influence [22]. Consequently, many Chinese cities have formulated a ’mega-event strategy’ and regard hosting large-scale events as a crucial means to increase cultural soft power, attract foreign investment, and drive urban development [6,23]. Various large-scale cultural events, such as World Expos, Olympics, and concerts hosted in Chinese cities, have significantly increased their status in cultural dissemination [6].

Among these global cultural events, international sports events (ISEs) have become the most favoured choice for Chinese cities for several reasons. First, ISEs have distinct levels and diversity [24,25], ranging from events with high requirements for comprehensive venues and socioeconomic conditions such as the Olympics and Formula 1 [26] to events with lower venue requirements such as marathons [27]. This allows cities to choose different types and levels of sports events based on their development conditions. In contrast, events such as World Expos and large concerts require better venue conditions [28], making them less viable options for cities at various levels. Second, for individual cities, ISEs have a significant spillover effect. Many international sports federations (ISFs) consider a city’s ability to host large sports events as a criterion for selecting host cities for events [29,30]. Therefore, cities that have hosted high-level sports events are more likely to be chosen as hosts for other single-sport events, further enhancing their status in sports cultural dissemination [31]. Additionally, sports stadiums/arenas can also be used for other cultural events, such as concerts and large exhibitions [32]. This has led many Chinese cities to consider hosting large-scale sports events as important development strategies to achieve their goals of enhancing urban soft power [6]. Therefore, this study aims to construct an external connectivity network of Chinese cities based on ISEs to reveal the spatiotemporal evolution of Chinese cities’ external connections from the cultural dimension, which may deepen our understanding of ISE-based WCNs and city-promotion pathways from the cultural dimension in the following two aspects:

First, ISEs are important global cultural phenomena that encompass broadness, diversity, and hierarchies [33]. Previous studies have focused mainly on individual ISEs and explored their impact on individual cities [34–36]. However, these studies have failed to explore the hierarchical nature of ISEs and reveal the spillover effects on other cities within the host country after they host the highest-level ISEs. Nevertheless, large ISEs, such as the Olympics, have had extensive impacts beyond the host city [37,38], as exemplified by the 2008 Beijing Olympics; the exceptional performance of Chinese athletes popularized niche sports around the country sparked increased interest in sports in many cities [39]. Therefore, investigating ISEs from the network perspective allows us to systematically analyse the spillover effects of mega-events and determine the interactions between different levels of events.

Second, through its national policy of reform and opening-up, China has become a typical country in the WCN that gradually emerged as a global leader in globalization in economic and political dimensions [7]. However, in terms of global cultural dissemination, China still occupies a relatively disadvantaged position [15,16]. To enhance the nation’s cultural soft power, Chinese cities have started using various means to increase their influence on cultural city networks [6,39]. However, owing to the diverse and specific nature of culture, its global dissemination has exhibited spatial and temporal patterns that are entirely distinct from those of economic and political globalization [26]. Taking China as an example can not only reveal the different paths that developing countries have followed to enhance their influence on various types of city networks but also provide a theoretical foundation and empirical reference for strategic planning in other developing countries.

Therefore, this study concentrates on the external connections of Chinese cities since 2008 based on the Olympic Games. By employing social network analysis, this research aims to analyse the evolving trends in the spatial patterns of external connections among Chinese cities from 2008 to the present. Additionally, this study integrates community detection analysis to determine the impact of the 2008 Beijing Summer Olympics and the 2022 Beijing Winter Olympics on the development of sports in China. Our research results not only reflect the distinct expansion patterns of Chinese cities using the ‘sports megaevent strategy’ but also highlight the gateway influence of two Beijing Olympic games, which may reveal various paths through which developing countries enhance their influence on city networks from different dimensions.

## 2 The essential role of sports events in soft power strategies in mainland China

In the premodern era, China was characterized by weakness and poverty and was frequently ridiculed by major global powers as the "sick man of East Asia". In seeking ways to address this issue, the Chinese government recognized the value of sports as a means of enhancing the nation’s soft power, shaping its image, and promoting diplomatic relations [6,39]. This process of development can be divided into three main stages (see note 1).

After the founding of the People’s Republic of China, the country’s sports underwent a period of "arduous initial stages." From 1949 to 1978, China primarily endeavoured to convey the enhancement of its citizens’ physical fitness and national soft power to the world through active participation in significant ISEs and by elevating athletes’ performance in these competitions. During this phase, Chinese athletes shattered a total of 18 world records across various sports events. However, the political disturbances during this period led to China’s withdrawal from the International Olympic Committee in 1958, significantly impeding its efforts to increase its soft power through engagement in major sports activities.

Between 1979 and 2008, China underwent reforms in its sports management system. The company aspired to improve its international image by enhancing athlete performance and hosting large-scale ISEs, thereby transforming international sports competitions into crucial platforms for external communication. Throughout this period, China consistently achieved the top ten places in the medal tally of numerous ISEs, particularly the Summer Olympic Games. Furthermore, China successfully hosted various ISEs, including the 1990 Beijing Asian Games and the 2008 Beijing Olympics, contributing to the substantial advancement of its sports sector. During this period, China’s progress in sports diffusion was reflected mainly in the breakthroughs in athletes’ performance rather than the continuous hosting of ISEs [40]. In essence, most of the ISEs hosted during this stage were regional events, such as the Beijing Asian Games and the Shanghai East Asian Games, with low frequency and limited number, typically hosting one ISE every 2–3 years. The hosting cities were highly concentrated in Beijing, Shanghai, and Guangzhou, with little participation from other cities, making it difficult to establish an effective sports diffusion network. Some scholars have noted that the reason China hosted ISEs during this stage was to demonstrate its ability to the International Olympic Committee (IOC) and ISFs, aiming to attract more high-level ISEs [40,41]. The true turning point was the hosting of the 2008 Beijing Olympics. After winning the bid in 2001, not only did Beijing itself begin building stadiums for the Olympics, but other cities hosting individual Olympic events (such as Qingdao) also started constructing event venues [37]. Furthermore, the strong influence of the Organizing Committee of Olympic Games (OCOG) of the Beijing Olympic Games promoted the hosting of some qualifying events in other Chinese cities [42], gradually positioning Chinese cities as important nodes in global sports diffusion.

The exceptional performance of Chinese athletes in various sports competitions and the successful organization of multiple international sports events showcased China’s developmental achievements to the world, reshaping its international image. This transformation elevated Chinese cities into vital nodes for the dissemination of sports culture and substantially enhanced their cultural soft power.

From 2008 to the present, the successful staging of the Beijing Olympics highlighted the transformative impact of major ISEs on urban development. Consequently, municipal governments at all levels within China became increasingly proactive in bidding for ISEs of varying magnitudes. By 2019, China had hosted a total of 200 ISEs, with numerous outstanding athletes achieving remarkable breakthroughs (e.g., Su Bingtian, Su Yiming). The stature of Chinese cities in the global sports and cultural communication network has continued to rise. The "major sports events" strategy has evolved into a pivotal developmental policy for Chinese cities and a crucial means for municipal governments to increase their standing.

The utilization of the ’Mega Sports Events’ strategy for urban marketing, aimed at cultivating urban images and attracting foreign investments, thereby elevating the city’s status within the WCN, has emerged as a pivotal driver in the developmental policy of Chinese municipalities [6]. However, extant investigations have focused predominantly on individual events and a single city. Specifically, scholars have focused primarily on (1) the impact of hosting major sports events on the host city itself—for example, studies have examined the impact of the 2008 Beijing Olympics and 2010 Guangzhou Asian Games on city marketing, the reshaping of urban/national images, and the dissemination of traditional Chinese culture [37,43]. (2) Integration into the sports-oriented WCNs of a single city [44]. For example, Chen’s use of the WCN approach was used to examine Shanghai’s position within the networks of six major ISEs, including snookers, and to explore the implications of large-scale sports events for the city’s development [45].

Nonetheless, these individual-event, single-city investigations fall short of providing a comprehensive depiction of the spatiotemporal dynamics and advancements of Chinese cities within the realm of sports event dissemination. These inquiries have two primary limitations: (1) they narrowly focus on the enhancement of the host city, neglecting the contributions of other cities. In essence, numerous cities also engage in the organization of lower-tier events, sponsorship, and broadcast activities associated with major international sports events, thereby becoming significant participants in WCNs and increasing their status [39]. (2) The study of individual cities fails to consider the continuity of China’s sports development process or the long-term evolutionary perspective of the impact of large-scale sports events on China’s soft power [19].

To address these gaps, this study analyses the spatial‒temporal evolution of the external connections of Chinese cities since 2008 during each Olympic Games. This approach is justified for several reasons: (1) The Olympics are the world’s largest and most influential ISEs, with the highest number of participants [28,33], and China’s competitive sports have always centred on the Olympics [46]. (2) The Olympics have a vast qualifying event system [47], and our study considers these qualifying events to be an important part of the city network surrounding the Olympic Games, thus allowing for a more comprehensive analysis of the impact of ISEs on various cities in China than did previous studies [26,29].

Additionally, as a critical secondary attribute of cities, the evolution of Chinese cities’ external connections during the previous Olympic games can effectively illustrate the characteristics and evolutionary paths of various cities in China that integrate into and even lead sports and cultural WCNs [20,48]. Shortly thereafter, it has been argued that the Olympic Games have a long-term impact on the host country [48], and the influence of sports events extends beyond the short term, affecting the economic, political, and cultural aspects of the country and city in the long term [49]. Consequently, longitudinal studies can better explore the long-term impact of ISEs on city/national soft power and analyse the specific paths of different-level cities embedded in WCNs at different times than traditional studies that focus on individual cities.

To summarize, the promotion of urban/national soft power through sports is highly important to China. As China’s economic development level has increased, hosting ISEs has become a vital component of the soft power strategy of the government at all levels. Nevertheless, the existing research on the relationships between China’s ISEs and city/national image building and soft power enhancement has concentrated solely on individual events or cities and has lacked a discussion of the spatial‒temporal evolution from a city network perspective. Therefore, this paper investigates the evolution of the external connections of Chinese cities during the Olympic Games beginning in 2008, which may deepen our understanding of China’s participation in sports globalization and glocalization while providing a practical reference for the soft power strategies of cities at all levels.

## 3 Data and methodologies

### 3.1 Samples and data resources

The sports event strategy of Chinese cities was initiated relatively late, with the 1990 Beijing Asian Games generally considered the first large-scale ISEs hosted in China [6]. However, from 1990 to 2008, China hosted a limited number of ISEs with low frequency. Except for the Women’s World Cup, there were no other global sports events, only regional events, which were highly concentrated in Beijing, Shanghai, and Guangzhou. Consequently, the external connections generated were limited to these three cities. Additionally, data on sponsors and broadcasters of ISEs in the 1990s were difficult to collect, making it impossible to fully construct the network of external connections generated by these events. After 2008, with the construction of numerous sports venues in various cities and the strong influence of the Beijing OCOG, both the number of ISEs and the number of host cities began to increase notably. Therefore, selecting the Olympic Games and Olympic qualifiers held after 2008 as the research objects can systematically demonstrate the evolution of the status of Chinese cities in international sports communication. It can also reveal the stages and paths through which they enhance their cultural influence and soft power through ISEs [26].

The data for this study are drawn from the official website of the International Olympic Committee and the official websites of various ISFs.

### 3.2 Intercity connections and activity values

According to Xue, intercity connections are forged through three main types of events during the process of organizing Olympic games, namely, power struggles, sponsorship activities and publicity activities, and each event may involve two specific events [26]. Power struggles may include competition for the rights to host Olympics and competition for athletes’ qualifications to participate in the Olympic games, while sponsorship activities may include the affairs of the TOP program and OCOG sponsorship program; additionally, publicity activities may include the affairs of torch relays and TV broadcasts (Table 1).

**Table 1:** Activity values of different events (revised from references 26).

Competition for the right to host the Olympic Games	Competition for athletes’ qualification to participate in the Olympic Games			TOP program	
Institutions	Activity values	Institutions	Activity values	Institutions	Activity values
IOC	5	IOC	5	IOC	5
Competition committee of host cities	4	OCOG	4	OCOG	4
Competition committee of cities that participate in the second round	2	ISFs	4	MNCs	4
Competition committee of cities that participate in the first round	1	Regional headquarters of the Olympic committee	3		
		NOCs	2		
		NSFs	1		
		Organizing Committee of qualifying Competitions	3,2,1		
OCOG sponsorship program		Torch relay		TV broadcast	
Institutions	Activity values	Institutions	Activity values	Institutions	Activity values
OCOG	5	OCOG	5	OCOG	5
OCOG partners	3	IOC	4	IOC	4
OCOG sponsors	2	Government of Athens	3	Rights-holding broadcasters	3
OCOG providers	1	Government of foreign cities	2	Non-rights-holding broadcasters	1
		Government of domestic cities	1		

**Fig 1:**
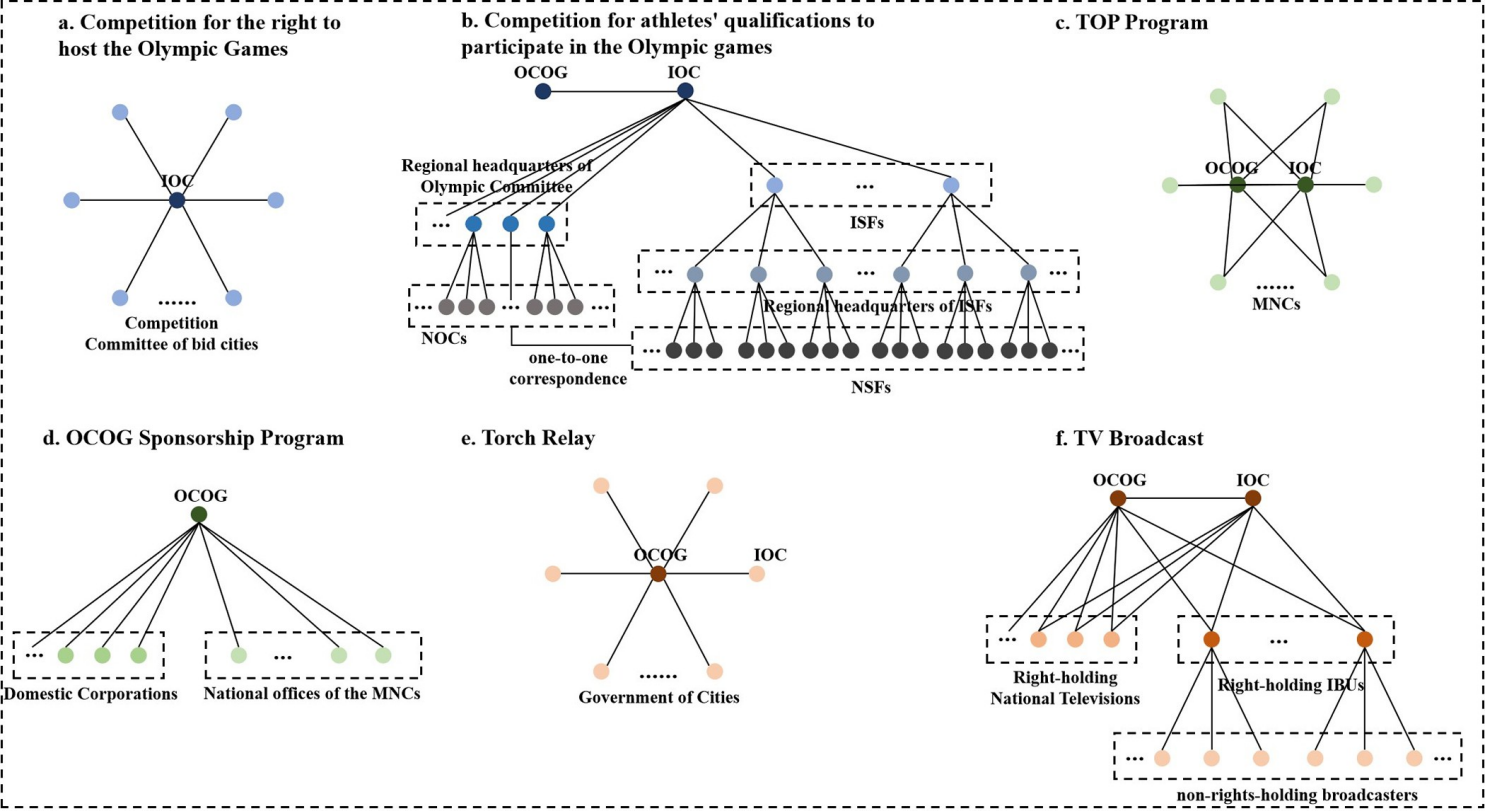
Intercity connections caused by the WOGs (revised from references 26,51).

Various actors are involved in these affairs, and the importance of each affair varies significantly. In different affairs, the actors are connected to each other, generating flows of people, information, and capital among different cities in the world. Therefore, a two-mode network model is created in which the actors are connected through the same affairs (Fig 1). based on Taylor’s promotion [50], different actors may assign diverse values according to their importance, and actors involved in the same affairs may interact closely with each other; however, no cross-affair connections occur during the organization process of ISEs [26].

Although some actors may play important roles in many affairs, the intercity connections caused by different affairs are independent, and the sum of connections between city *a* and city *b* in the six subnetworks may be considered their connections generated by the Olympic Games.

According to Xue, the specific connections generated by six affairs are as follows [26]. However, Xue’s promotion overlooked Olympic-related qualifying events. In essence, qualifying events are extremely important in the organization of the Olympics. The eligibility of athletes in almost all Olympic sports is determined through these qualifying events [47]. In addition, organizing domestic athletes to compete in those qualifying events is crucial for both National Olympic Committees (NOCs) and National Sports Federations (NSFs) [52]. This means that during the organization of the Olympics, the organizing committees of qualifying events established very close connections with various levels of Sports Federations. Therefore, neglecting qualifying events within the intercity connections framework would render the organizational system of the Olympics highly incomplete. Therefore, our attempts may involve those actors in the subnetwork (Fig 1).

Additionally, significant differences are found in the roles that different actors play in various areas. Different actors are assigned different values based on their importance in events (Table 1), and the sum of the values of all actors in a city is considered the value of the city in that activity [26,50].

### 3.3 Methodologies

We aimed to identify both vertical and horizontal connections between different actors [53]. therefore, social network analysis and complex network analysis were used to explore the characteristics of the intercity connections. Social network analysis is a commonly used method for studying cities worldwide. This method accurately demonstrates the intercity connections between different cities and reveals the structural characteristics of the network through various network indicators [3,53]. This provides data support for the analysis in this study. Specifically, this study requires the integration of assigned values from different entities to determine the connectivity values between different cities.

$$C_{ab,j}=\sum_{m,n}V_{ma,j}*V_{nb,j}\;C_{ab}=\sum_{j}C_{ab,j}\;C_{a}=\sum_{b}C_{ab}$$ (1)

where *V*_*ma*,*j*_ refers to the activity values of actor *m* in city *an* in event *j*, ***V***_***nb*,*j***_ refers to the activity values of actor *n* in city *b* in event *j*, *C*_*ab*,*j*_ represents the directed connectivity between cities *a* and *b* in event *j*, *C*_*ab*_ refers to the connections between *a* and *b* in all events, and *C*_*a*_ refers to the connectivity of city *a*.

Furthermore, calculating the betweenness centrality of Beijing in each Olympic Games is necessary to identify the direct and indirect connections between various cities in China and foreign cities. This analysis aimed to summarize the mechanisms involved in the transformation of direct and indirect connections.


$$Betweennes\;s(u)=\underset{s\neq u\neq t}{\sum }\frac{p(u)}{p}$$ (2)

In this context, *u* represents the node under consideration for calculation, *p* denotes the total number of shortest paths between nodes *s* and *t*, and *p(u)* represents the number of shortest paths between *s* and *t* that pass through node *u*.

Finally, we employ community detection methods, specifically the Infomap algorithm and F-B algorithm, to analyse the formation of regional subnetworks between the 2008 and 2022 Beijing Olympic Games [54]. This analysis thus explores the transformation of roles and status improvement for Chinese cities in ISEs’ dissemination over a span of 14 years.

### 4 Expansion of city nodes: Spatial‒temporal evolution of Chinese cities’ activity values since 2008

**Fig 2:**
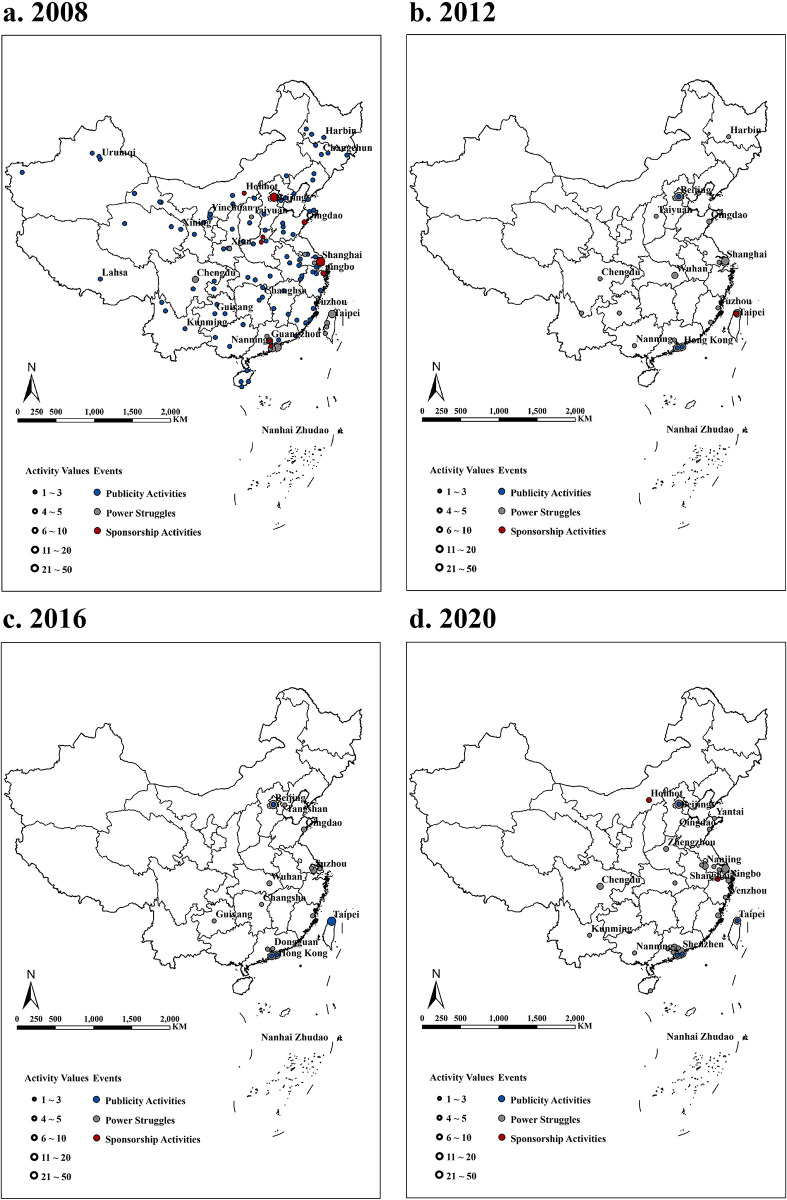
Activity levels of Chinese cities in four Summer Olympic games (The maps we used in Fig 2 are sourced from Ministry of Natural Resources of the People’s Republic of China (http://bzdt.ch.mnr.gov.cn/), the approval number is GS2020(4619)).

Providing event broadcasting, engaging in sponsorship activities, and hosting ISEs are three crucial components in promoting the development of sports. Since 2008, an increasing number of cities in China have regarded the participation in or hosting of ISEs as important means of enhancing their cultural influence and soft power [39,42]. In terms of power struggles during the 2008 Olympics, only 10 cities in mainland China hosted Olympic qualifying events, and these events were mostly concentrated in economically developed central cities such as Beijing, Shanghai, Guangzhou, and Chengdu, indicating a clear trend of agglomeration. Following the end of the Beijing Olympics, driven by corresponding policy incentives and the continuous improvement of urban infrastructure in Chinese cities at all levels, local governments at all levels eagerly embraced hosting large-scale sports events as a means to create advanced demand and promote urban development [6,55]. The strong demand for the "globalization" of various ISFs further increased the trend of Chinese cities hosting Olympic qualifying events (13 cities in 2012, 14 cities in 2016, and 24 cities in 2020) [46]. Moreover, host cities have expanded from first-tier cities to provincial capitals and second-tier cities. Cities such as Kunming, Dongguan, Foshan, and Yantai capitalize on their own infrastructure conditions and host international badminton and sailing tournaments (Fig 2). These endeavors have further enhanced the influence of Chinese cities in the realm of sports events.

In addition to the increase in the number of cities, the range of Olympic qualifying events hosted by China has gradually expanded. During the Beijing Olympics in 2008, China primarily hosted preliminary rounds of advantageous events such as badminton, table tennis, and diving. However, driven by the alignment of interests (including the city government, corporations and NGOS) at the national and city levels, regional and international qualifying events for various Olympic sports, including basketball, athletics, field hockey, and judo, began to select cities of different levels in China as host locations. In fact, the promotion of the "events globalization" strategy by ISFs and the attraction measures taken by Chinese city governments for ISEs are two main factors that contributed to this expansion. On the one hand, the fundamental mission of ISFs’ headquarters is to globalize their respective sports and enhance their influence [32,46]. Therefore, selecting emerging cities with mature infrastructure as hosts for newly added international events is an important means of promoting the popularization of sports [30]. The rapid development of Chinese cities in recent years, coupled with the relatively low popularity of certain individual sports domestically, has led to an increasing number of ISFs seeking to see international sports events hosted by Chinese cities. On the other hand, international sports events provide significant marketing opportunities for cities, allowing them to reshape their image, attract foreign investment [37,56], and generate substantial forwards demand [55,57], resulting in rapid development within a short period. This positive effect is highly attractive to sitting city officials [6]. Therefore, under the leadership of city governments, many Chinese cities have made hosting international sports events an integral part of their urban development strategies. Through the interaction of these promotion-attract mechanisms, cities at different levels in China have significantly elevated their status within the ISE-based network.

In the context of the Winter Olympics, the cities in China that participated in the network presented significant clustering characteristics. Apart from Taipei, Hong Kong, and Macau, as well as Hangzhou, owing to its inclusion in the Olympic Committee’s sponsorship activities, only Beijing and some cities in northeastern China (such as Harbin and Changchun) were involved in the construction of city networks based on the Olympic Games during the three editions of the Winter Olympics from 2010 to 2018. This weakness can be attributed to two main factors. First, economically developed cities along the southeastern coast of China have a hot climate and lack the climatic and population foundations for winter sports [58]. Second, in the vast northern regions of China, including the northwest and northeast regions, climate conditions are highly suitable for winter sports, but owing to the high requirements of winter sports venues, substantial investments are needed to construct adequate training and competition facilities [59]. The relatively underdeveloped enterprises in the northern regions of China, coupled with the financial pressures faced by city governments [60], pose challenges in hosting large-scale winter sports events in these areas. Consequently, only a few economically prosperous cities in northeastern China have decided to hold certain events, while the majority of Winter Olympics qualifiers have been concentrated in the capital city, Beijing.

This does not imply that China has not made efforts to increase its soft power in winter sports. Table 2 shows that the activity values of Chinese cities in power struggles have continuously increased. This change primarily reflects China’s increased participation in various individual Winter Olympics events since 2010. The focus has shifted from an early emphasis on ice skating and skiing events to breakthroughs in curling, ice hockey and bobsleigh/skeleton events during the 2022 Beijing Winter Olympics. As a result, China’s cultural influence on winter sports has significantly improved. However, owing to climate conditions and the limited popularity of winter sports, China has yet to achieve diffusion from core cities (such as Beijing) to first-tier and second-tier cities in the realm of winter sports.

**Table 2:** Activity levels of Chinese cities during the four Winter Olympic Games.

	All			Power Struggles			Sponsorship Activities			Publicity Activities		
	City	Values	Proportion	city	Values	Proportion	city	Values	Proportion	City	Values	Proportion
**2010 Vancouver**												
1	Taipei	10	1.18%	Beijing	6	1.14%	Taipei	4	3.28%	Taipei	3	1.52%
2	Beijing	9	1.07%	Taipei	3	0.57%	Hangzhou	1	0.82%	Beijing	3	1.52%
3	Hong Kong	3	0.36%	Hong Kong	3	0.57%				Hong Kong	1	0.51%
4	Changchun	1	0.12%	Changchun	1	0.19%						
5	Hangzhou	1	0.12%	Harbin	1	0.19%						
6	Harbin	1	0.12%									
		844	2.96%		525	2.67%		122	4.10%		197	3.55%
**2014 Sochi**												
1	Beijing	10	0.91%	Beijing	7	1.11%				Beijing	3	0.80%
2	Taipei	5	0.46%	Taipei	4	0.63%				Taipei	1	0.27%
3	Hong Kong	4	0.36%	Hong Kong	3	0.48%				Hong Kong	1	0.27%
		1096	1.73%		630	2.22%					373	1.34%
**2018 Pyeongchang**												
1	Beijing	14	1.27%	Beijing	11	1.68%	Hangzhou	4	2.76%	Beijing	3	0.98%
2	Taipei	5	0.45%	Taipei	4	0.61%				Taipei	1	0.33%
3	Hong Kong	4	0.36%	Hong Kong	3	0.46%				Hong Kong	1	0.33%
4	Hangzhou	4	0.36%	Changchun	3	0.46%						
5	Changchun	3	0.27%									
		1106	2.71%		655	3.21%		145	2.76%		306	1.63%
**2022 Beijing**												
1	Beijing	84	8.69%	Beijing	17	2.60%	Beijing	53	38.69%	Beijing	14	7.95%
2	Shanghai	8	0.83%	Zhangjiakou	3	0.46%	Shanghai	8	5.84%	Yanqing	1	0.57%
3	Hangzhou	4	0.41%	Yanqing	3	0.46%	Hangzhou	4	2.92%	Zhangjiakou	1	0.57%
4	Zhangjiakou	4	0.41%				Huerhaote	3	2.19%			
5	Yanqing	4	0.41%				Xiamen	3	2.19%			
6	Huerhaote	3	0.31%				Qingdao	2	1.46%			
7	Xiamen	3	0.31%				Putian	1	0.73%			
8	Qingdao	2	0.21%				Hefei	1	0.73%			
9	Zhuhai	1	0.10%				Zhuhai	1	0.73%			
10	Putian	1	0.10%									
11	Hefei	1	0.10%									
		967	11.89%		654	3.52%		137	55.47%		176	9.09%

## 5 Indirect-to-direct: External connections of Chinese cities since 2008

As Table 3 shows, in addition to the Olympics hosted in China, Chinese cities have established connections with cities in only three regions, namely, Europe, Asia and North America, in the remaining Olympic games.

**Table 3:** External Connections of Chinese Cities in the Four Summer Olympic Games.

Regions	Power Struggles		Sponsorship Activities		Publicity Activities	
	Connectivities	Proportion	Connectivities	Proportion	Connectivities	Proportion
**2008 Beijing**						
China	0	0.00%	356	59.33%	555	51.29%
Africa	0	0.00%	0	0.00%	55	5.08%
Asia (Except China)	52	15.29%	32	5.33%	145	13.40%
Europe	268	78.82%	116	19.33%	162	14.97%
North America	20	5.88%	96	16.00%	115	10.63%
South America	0	0.00%	0	0.00%	10	0.92%
Oceania	0	0.00%	0	0.00%	40	3.70%
All	340	100.00%	600	100.00%	1082	100.00%
**2012 London**						
China	0	0.00%	0	0.00%	0	0.00%
Africa	0	0.00%	0	0.00%	0	0.00%
Asia (Except China)	72	22.78%	0	0.00%	3	3.57%
Europe	244	77.22%	56	100.00%	81	96.43%
North America	0	0.00%	0	0.00%	0	0.00%
South America	0	0.00%	0	0.00%	0	0.00%
Oceania	0	0.00%	0	0.00%	0	0.00%
All	316	100.00%	56	100.00%	84	100.00%
**2016 Rio de Janeiro**						
China	0	0.00%	0	0.00%	0	0.00%
Africa	0	0.00%	0	0.00%	0	0.00%
Asia (Except China)	72	31.03%	0	0.00%	9	100.00%
Europe	160	68.97%	0	0.00%	0	0.00%
North America	0	0.00%	0	0.00%	0	0.00%
South America	0	0.00%	0	0.00%	0	0.00%
Oceania	0	0.00%	0	0.00%	0	0.00%
All	232	100.00%	0	0.00%	9	100.00%
**2020 Tokyo**						
China	0	0.00%	0	0.00%	3	8.33%
Africa	0	0.00%	0	0.00%	0	0.00%
Asia (Except China)	68	18.28%	32	28.57%	21	58.33%
Europe	296	79.57%	80	71.43%	12	33.33%
North America	8	2.15%	0	0.00%	0	0.00%
South America	0	0.00%	0	0.00%	0	0.00%
Oceania	0	0.00%	0	0.00%	0	0.00%
All	372	100.00%	112	100.00%	36	100.00%

In various Olympic games, China has actively engaged in sponsorship and publicity activities. However, in these two events, the external connections of Chinese cities differ significantly from those of the Olympics. Specifically, in the event of sponsorship activities, as the host nation, China has an OCOG sponsorship program targeting domestic enterprises, resulting in numerous domestic connections [26]. In other Olympics, Chinese cities only establish connections with the current OCOG and IOC when local enterprises become TOP (The Olympic partner) sponsors. The threshold for TOP sponsorship is extremely high, with fewer than ten corporations achieving this status each Olympic period [47]. Therefore, in non-Chinese-hosted Olympics, cities with TOP sponsors connect directly with the OCOG and IOC, but only one or two Chinese cities typically become significant nodes in sponsorship activities (i.e., Alibaba in Hangzhou, Yili in Hohhot) (Table 3).

In publicity activities, the OCOG needs to provide broadcasting signals to rights-holding broadcasters [52]. Consequently, during the 2008 and 2022 Beijing Olympics, Beijing established connections with numerous cities hosting these broadcasters and formed direct connections with many Chinese cities through the torch relay [61]. In comparison, during the Olympics not hosted by China, only the rights-holding broadcaster formed a direct connection with the OCOG and IOC. Historically, CCTV has been the sole rights-holding broadcaster in China for all Olympic games, whereas Chinese cities have not achieved broadcasting rights coverage in other Asian regions (some official broadcasters hold broadcasting rights for all national Olympic games within their respective regions, whereas others hold broadcasting rights only for their own country’s Olympic games; CCTV belongs to the latter category) [47]. As a result, Chinese cities are still in the stage of actively integrating themselves into these two types of events rather than assuming a leading role.

**Fig 3:**
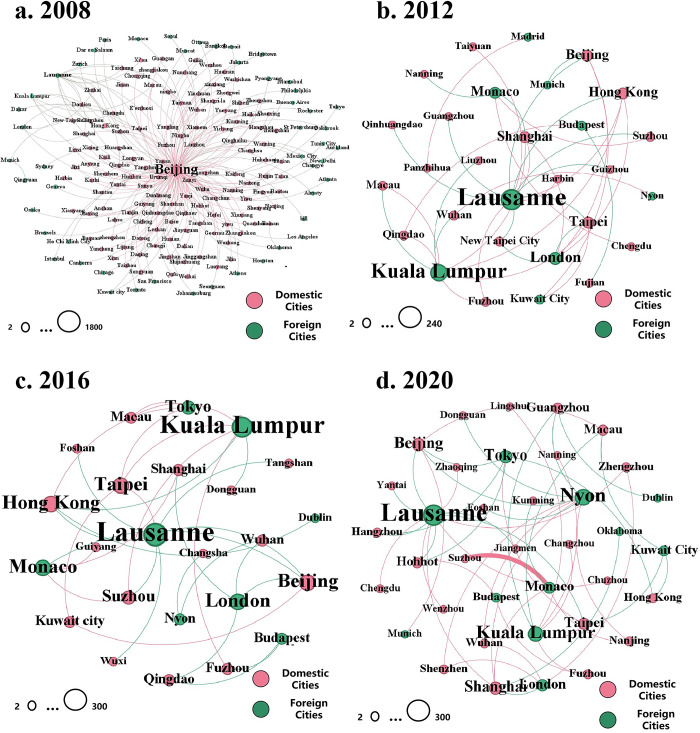
External connections of Chinese cities in four summer Olympic games.

However, in terms of power struggles, the influence of Chinese cities has been continuously expanding, and China has increasingly become a "hotspot region" for hosting ISEs [27]. In every Olympic Games, Beijing, as a city with all NSFs, establishes direct connections with the IOC and various ISFs because the Olympic participation lists are confirmed, whereas other cities can only form direct connections with the cities hosting individual ISFs by organizing qualifying events. From an evolutionary perspective [26], during the 2008 Beijing Olympics, most Chinese cities only had indirect connections with foreign cities and a relatively low position in the city network (Fig 3). With the "promote-attract" interactions between ISFs and various levels of the Chinese government, an increasing number of Chinese cities have started to host Olympic qualifiers and establish direct connections with core cities in power struggles [6]. As a result, the intermediary centrality of Beijing as an intermediary city has been decreasing (Table 4), and direct connections between Chinese cities and other cities have significantly increased.

**Table 4:** Betweenness centrality of Beijing in four summer Olympic games.

	2008	2012	2016	2020
Beijing	0.27	0.10	0.06	0.05

Analysing the external connections of Chinese cities through the three types of events reveals that the connections in each event are fundamentally independent due to the involvement of different actors (Fig 1). Although hosting the Olympics domestically can somewhat enhance indirect connections between domestic cities and foreign cities, the lack of direct connections means that these indirect links do not significantly elevate any particular city’s status in ISE-based WCNs [27].

In terms of individual cities, in addition to Beijing, which was the location of all Chinese NSFs and CCTV, Beijing established direct connections with other cities during every Olympics. Other cities must host qualifying events or become TOP sponsors to form direct connections with foreign cities. During the 2008 Olympics and the 2022 Olympics, Beijing, as the city with OCOG, formed direct connections with many foreign cities through sponsorship and publicity activities. Only a few cities hosted qualifying events in 2008–2022. Beijing established direct connections with many Chinese cities through torch relays and the OCOG sponsorship program [26]; this dual linkage made Beijing a crucial hub for connections between Chinese and foreign cities.

However, during the 2012, 2016, and 2020 Summer Olympic Games, the "major event strategy" of different Chinese city governments was highly effective [6,39]. An increasing number of ISEs are hosted in China, with the number of cities hosting Olympic qualifying events increasing from 10 in 2008 to 24 in 2020. Additionally, Chinese companies such as Alibaba and Yili became TOP sponsors, leading to more direct intercity connections between foreign cities (mainly cities with IOC and ISF headquarters) and various Chinese cities. The decreasing betweenness centrality of Beijing indicates that although it remains the Chinese city with the most foreign connections, other Chinese cities no longer rely on Beijing as a hub for indirect connections with other cities. Consequently, Beijing’s roles as a "gateway city" and "hub city" are continuously weakened (Table 4).

**Fig 4:**
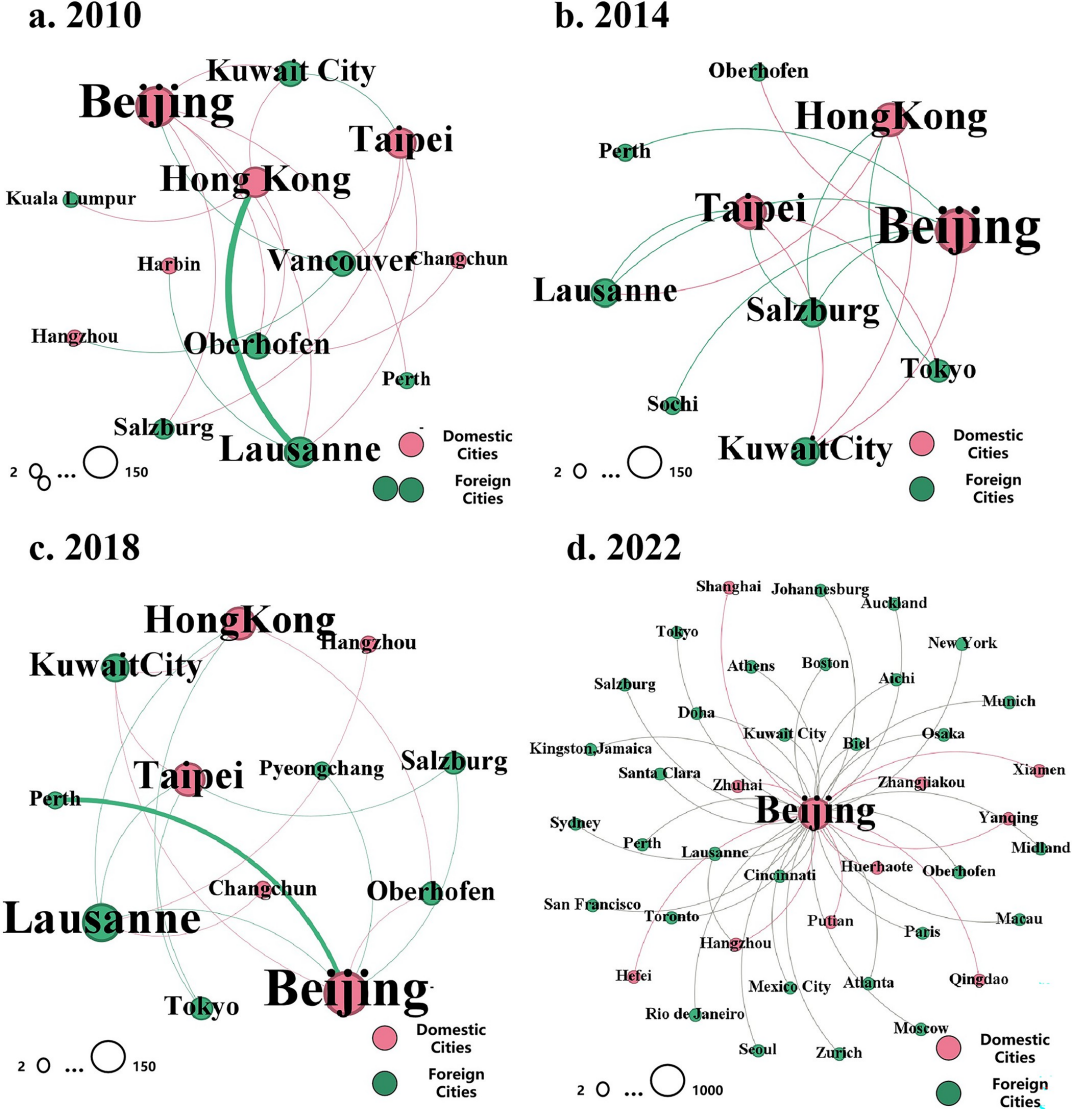
External connections of Chinese cities in the four Winter Olympic games.

In the city network based on the Winter Olympics, except the 2022 Beijing Winter Olympic Games, only a few cities can generate external connections in WOGs, and Chinese cities with national/regional headquarters of ISFs, including Hong Kong, Beijing, and Taipei, always generate more external connections with foreign cities (Fig 4). In recent years, newly added ISEs have been concentrated primarily in Beijing. However, owing to the inherent weaknesses of winter sports, China’s efforts to increase its influence on winter events have focused mainly on improving the competitiveness of athletes in various disciplines. This includes cultivating more athletes who meet Olympic qualification standards and achieving better results in various competitions. After several years of effort, China achieved full coverage of participating events in the 2022 Winter Olympics and obtained 9 gold, 4 silver, and 2 bronze medals, marking its best performance, with a third-place ranking in the medal tally. This achievement significantly enhanced the influence of winter sports in China, laying a solid foundation for the widespread promotion of winter sports and the hosting of more winter events by establishing a solid base of support among the public.

### 6 From embeddedness to dominance: Different subnetworks in the 2008 and 2022 Olympic Games in Beijing

Bale described in his research that the diffusion of a sport in a specific region is influenced not only by the region’s socioeconomic conditions but also by the impact of the sport itself within the region [32,62]. Similarly, Zhang highlights that an alliance of interest, composed of government, businesses, and nongovernmental organizations, initially assesses the benefits and drawbacks of hosting ISEs in a city [55]. If the alliance determines that the event lacks sufficient influence to shape the city’s image and attract foreign investment, it will be challenging to successfully implement the event. Hence, the level of ISEs and the popularity of individual sports within the local market are regarded as significant factors influencing the decision-making process of a city’s interest alliance [57].

**Fig 5:**
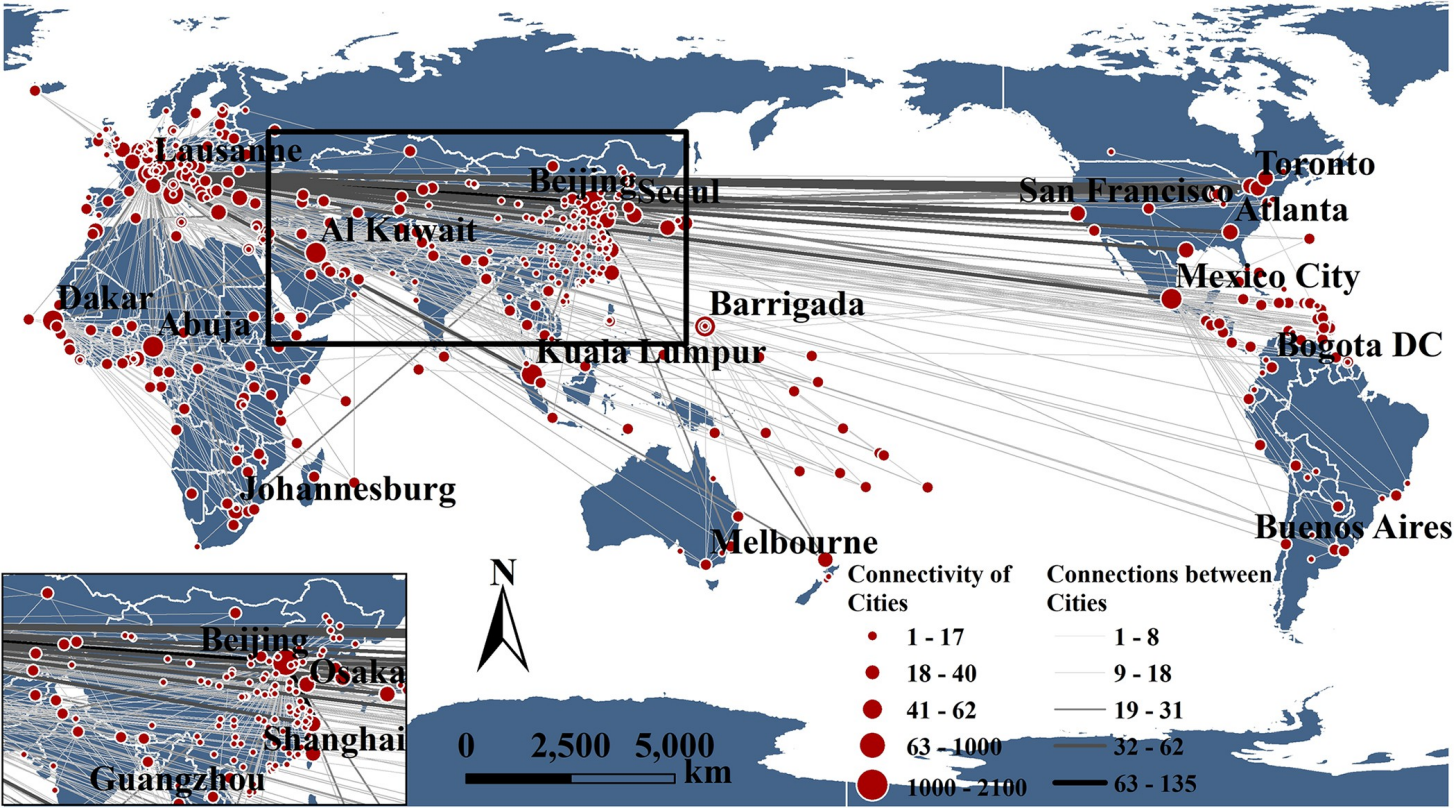
WCN and communities based on the 2008 Beijing Olympic Games ((The map we used in Fig 5 is sourced from Ministry of Natural Resources of the People’s Republic of China (http://bzdt.ch.mnr.gov.cn/), the approval number is GS2016(1666)).

Before the 2008 Olympic Games, only economically developed cities such as Beijing and Shanghai hosted Olympic-qualifying events, with the intention of laying the foundation for hosting large-scale comprehensive sports events in the future [40]. During the Beijing Olympic Games, more than 100 cities in China were incorporated into a subnetwork centred on Beijing (Figs 3 and 5), establishing numerous indirect connections with foreign core cities (albeit lacking direct connections) (Fig 5). Moreover, the tremendous success of the Olympic Games itself ignited a sense of national pride among the Chinese people. Several less popular sports (such as weightlifting and fencing) have gained immense popularity because of the emergence of national Olympic champions [39]. Since the Beijing Olympic Games, various levels of city government in China have recognized the significant enhancement of city status and the substantial developmental impetus brought about by hosting major international sports events [6]. Consequently, they have begun to leverage their existing infrastructure conditions to seek opportunities to host ISEs at different levels. Owing to this stage of rapid development of ISEs in China, the popularity of such sports has increased.

The hosting of the 2008 Beijing Olympic Games served as a catalyst for increasing China’s international image and status, highlighting achievements in the country’s development since the era of reform and opening up [37]. The event marked an important milestone and prompted numerous cities to seek opportunities to host ISEs [55]. As a result, the indirect connections among Chinese cities within the sports event network gradually transformed into direct connections (Fig 3), facilitating China’s rapid integration into WCNs based on ISEs. This process significantly enhanced the position of various Chinese cities in terms of international sports dissemination.

**Fig 6:**
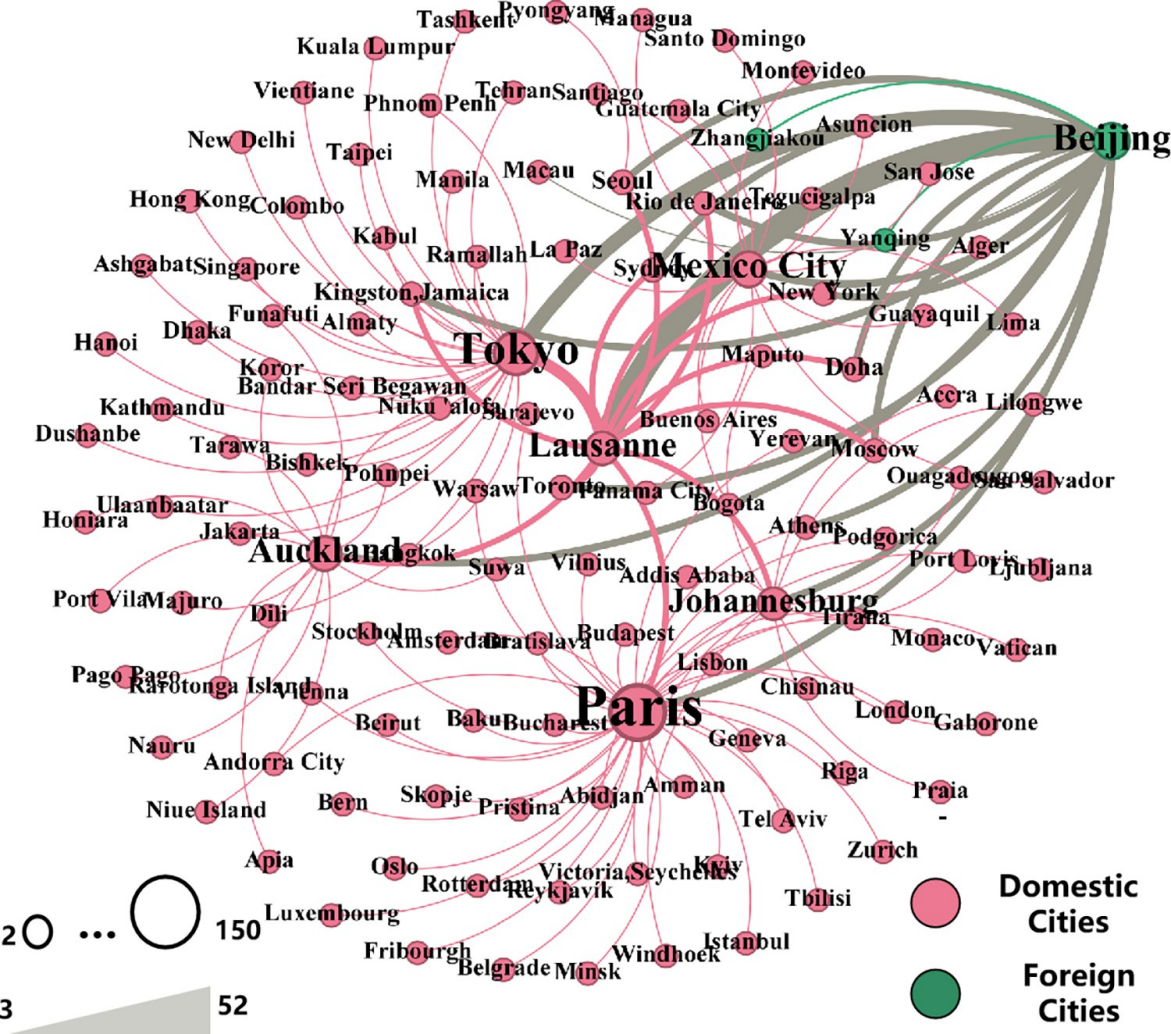
External connections of Chinese cities based on publicity activities during the 2022 Olympic games.

The 2008 Beijing Olympic Games accelerated the integration of Chinese cities into WCNs based on ISEs, whereas the 2022 Beijing Winter Olympics marked the beginning of Chinese cities gradually projecting their own values and enhancing their leadership capabilities on the global stage. Prior to 2022, mainland China lacked the presence of ISFs’ headquarters or regional headquarters and did not have access to regional broadcasting rights for corresponding events. As a result, Chinese cities had to promote themselves as significant participants in the ISE system by hosting more higher-level ISEs than before and becoming high-level sponsors [26,27]. However, in 2022, China achieved comprehensive dominance in publicity activities (Fig 6) through significant advancements in broadcasting technology and broader coverage of broadcasting rights. On the one hand, CCTV utilizes advanced technologies such as high-speed cameras and 4K broadcasting to create spectacular ice and snow extravaganza, delivering exceptional audiovisual effects to audiences worldwide. On the other hand, the Beijing Winter Olympics achieved historical global-scale broadcasting in several countries in South America and Africa for the first time. Beijing has close connections with all regional-core cities (Fig 6), highlighting the significance of the Beijing Winter Olympics in promoting the global popularity of winter sports [51]. This accomplishment also facilitated the enhancement of Chinese cities’ leadership capabilities at specific sports events.

Finally, the successful hosting of the Beijing Winter Olympics has not only significantly improved China’s urban leadership capabilities but also greatly increased the number of Chinese participants in winter sports [59]. The 2022 Beijing Winter Olympics may, similar to the 2008 Beijing Olympics, become a pivotal event for various cities in China, marking a turning point for large-scale international winter sports competitions in urban settings.

## 7 Conclusion

This study combines theories of world cities and social network analysis methods to investigate the external connections of Chinese cities during each Olympic Games since 2008. The findings of this study are as follows:

Since 2008, Chinese cities have been actively integrated into WCNs based on ISEs. This proactive involvement has led to an increasing influence, with a diffusion trend observed from first-tier cities to second-tier cities and provincial capitals. The fundamental reason behind this trend lies in the promotion of ISFs and the attractive measurement of Chinese cities’ governments. On the one hand, to promote the globalization of sports events, ISFs are trying to select Chinese host cities considering China’s rapid development in recent years and the relatively lower popularity of certain sports domestically [37,63]. On the other hand, as ISEs offer significant opportunities for city marketing, urban image reshaping [34], and attracting foreign investments [55], many Chinese cities have incorporated hosting ISEs as an integral part of their urban development strategies [6]. As a result, Chinese cities at different levels have hosted multiple Olympic-related qualifying events, leading to a transformation in Chinese cities in ISE-based WCNs. Before 2008, indirect connections were predominant, with Beijing acting as an intermediary city. However, with the hosting of these qualifying events, direct connections have become the primary mode of engagement. The evolution of this spatial pattern and the changes in city connections reveal the long-term spillover effect of large-scale ISEs.

From the perspective of ISE-based WCNs, existing research has focused primarily on the evolution of overall spatial patterns and hierarchical structures, uncovering the trend of nonwesternization of such networks and the rising prominence of Asian cities [35,51,64]. This study, through a case analysis of Chinese cities, argues that this spatiotemporal evolution trend may originate from the nonwesternization of hosting rights for major sports events such as the Olympics. First, hosting the Olympics serves as a significant platform for the host country/cities to show their development achievements[38,65], demonstrating the host nation’s ability to organize large-scale events [48]. This makes ISFs more inclined to place new events in the host cities of Olympics or other cities within the host country because of the ‘promotion-attract’ effect [29]. Second, the successful bid to host the Olympics increases the popularity of individual sports within the host country, enhancing training conditions and enabling athletes to qualify for more sports [33,47,52], further increasing the status of various cities within the host country. Beyond China, this spillover effect may also be helpful for explaining the increasingly important role of Asian countries such as Japan and South Korea [35,42,59] and their major cities in global sports diffusion. The effect of the Olympics on enhancing the soft power of these rapidly developing economies has not quickly become apparent, but over time, this spillover effect ultimately impacts the overall structure of ISE-based WCNs.

Additionally, this study revealed that the 2008 Beijing Olympics and the 2022 Beijing Winter Olympics were pivotal events that promoted the development of sports diffusion in China. Through the indirect external connections established during the 2008 Beijing Olympics, many Chinese cities realized the tremendous impact of ISEs and began seeking opportunities to host various levels of competition. However, owing to the lack of ISA headquarters, Chinese cities are still primarily in the phase of actively integrating into WCNs based on ISEs. Nevertheless, the successful hosting of the 2022 Beijing Winter Olympics marked the first comprehensive dominance of Beijing in publicity activities, which has provided Chinese cities with a pathway to gradually enhance their leadership capabilities by starting with publicity activities. This result indicates that, in the cultural dimension, the implementation of "gateway events" may be more important than "gateway cities" in promoting the enhancement of cultural soft power in developing countries such as China. On the one hand, the development of Chinese cities in economic and political dimensions is initiated by specific cities, gradually expanding outwards through multiple gateway cities (such as Shanghai, Guangzhou, and Hong Kong) and increasing their urban status [4,5,66]. However, in this study, Chinese cities needed to rely on Beijing for their external connections only in the initial stage. Since 2012, there has been a significant increase in direct connections among cities of different levels, and various city governments have begun to host multiple ISEs through their own initiative [6]; the "event-led" strategy at the urban scale has greatly reduced the emergence and presence of gateway cities. On the other hand, the hosting of high-level global cultural events in the host country allows local government officials to observe not only the significant driving force of global cultural events for urban development [6,57] but also international cultural associations to observe the host country’s achievements and ability to successfully host large-scale events. Consequently, more cities in the host country are selected as host cities for cultural events, resulting in a synergetic and stimulating effect on lower-level events, thereby promoting the enhancement of the country’s soft power.

In summary, this study examines the long-term effects of hosting major sports events on different cities within the host country from the perspective of intercity connections and determines the spatial‒temporal expansion patterns of Chinese cities from a cultural perspective. This study reveals the spillover effects of high-level ISEs on lower-level events, thus offering new insights for globalization practices in sports. However, this study also has several limitations. First, it focuses solely on Chinese cities and the spillover effect of their large-scale sports events, ignoring the long-term processes by which other developing countries, such as Japan, South Korea, South Africa, and Brazil, enhance their national soft power through ISEs and their impacts [34,42]. Additionally, this study considers only international-level sports events in China, neglecting lower-tier events such as regional qualifiers and national qualifiers. Therefore, this study calls for two types of research: (1) network studies that focus on China and various levels of sports events to gain a deeper understanding of the overall structure of ISE-based WCNs in China and (2) research on the evolutionary characteristics of ISE-based WCNs in other Olympic host countries, which could provide more understanding of the diverse paths through which countries enhance their soft power via ISEs and their impact on the overall structure of global sports diffusion.

### Notes

1. The divided period of sport development in China came from the article ‘70 years of sports in China’ written by Gou Zhongwen (Former director, General Administration of Sport of China).

https://www.sport.gov.cn/n10503/c927997/content.html#:~:text=%E6%96%B0%E4%B8%AD%E5%9B%BD%E4%BD%93%E8%82%B270%E5%B9%B4,%E8%B5%B0%E5%90%91%E4%B8%96%E7%95%8C%E7%9A%84%E5%8E%86%E5%8F%B2%E3%80%82.

## Supporting information

S1 File Data of external connections based on Summer Olympics. (XLSX)

S2 File Data of external connections based on Winter Olympics. (ZIP)

## Abbreviations

2: We use some abbreviations in the article to prevent us from repeating the names of organizations or terminologies; here is the list of abbreviations
WCN: world city network NGO—non-governmental organization
ISF: International Sports Federation ISE–International sports event
TOP: The Olympic Partner Program MNC–Multinational Corporation
IOC: International Olympic Committee NOC–National Olympic Committee
NSF: National Sport Federation OCOG—Organizing Committee of Olympic Games
APS: Advance Producer Service WSC–World Sports City
